# A study on the evolution of tripartite collaborative prevention and control under public health emergencies using COVID-19 as an example

**DOI:** 10.1038/s41598-024-53601-3

**Published:** 2024-02-07

**Authors:** Liu Mingyue, Shen Wei, Xin Zhang

**Affiliations:** School of Civil Engineering and Architecture, Wuhan Institute of Technology, Wuhan, 430074 China

**Keywords:** Applied mathematics, Public health

## Abstract

The problem of repeated epidemic fluctuations in the normalized prevention and control stage is revealed by data from January 20, 2020, to January 30, 2023. In order to improve the collaborative response of the public and government departments to public health emergencies and avoid repeated fluctuations of the epidemic, a tripartite evolutionary game model of the public, local government, and central government departments is constructed, focusing on the evolutionary paths and evolutionary stabilization strategies of the three subjects, and the influence of each element on the evolutionary results is simulated by numerical simulation in Matlab, and based on the inadequacy of the static reward and punishment mechanism, a dynamic Based on the shortcomings of static reward and punishment mechanism, dynamic reward and punishment mechanism is introduced to control the stability of the evolving system. The study shows that (1) with the increase of the initial willingness of the three parties, the rate of the public choosing the discretionary flow strategy slows down, and the collaborative prevention and control process can be accelerated. (2) The reward and punishment mechanism of central government departments has a positive incentive effect on the local government's strict prevention and control and the public's conscious isolation. Appropriately increasing rewards, formulating reasonable subsidy strategies, and increasing penalties for violations are conducive to the overall optimization of the system, and the punishment mechanism is most sensitive to the regulation of the public's discretionary mobility behavior. (3) Government departments' prevention and control costs can influence their enthusiasm for strict prevention and control and real-time supervision. Reducing the human resources cost, time cost, and financial cost of prevention and control is conducive to government departments performing their duties more responsibly. (4) The static punishment mechanism fails to make timely adjustments according to the strategy choice of each actor. It cannot control the stability of the evolving system. In contrast, the dynamic punishment mechanism considers the punishment parameters to link the casual isolation rate with the lenient prevention and control rate, which can effectively control the system's fluctuating instability and is the system's stability control strategy. Finally, combining theoretical and simulation analysis, management suggestions are made for controlling repeated fluctuations of the epidemic in practice, and the research limitations of this paper are explained.

## Introduction

In early 2020, a new coronavirus (COVID-19, hereafter referred to as "new coronavirus") broke out. It spread rapidly worldwide, causing significant loss of life and property in countries worldwide^1^. By January 30, 2023, more than 480 million cases of NCC have been diagnosed worldwide, resulting in more than 6 million deaths. China is a "model country" in responding to the NCC epidemic. Still, after entering the normalized stage of epidemic prevention and control, there are a series of coordinated prevention and control problems, such as the public's non-compliance with epidemic prevention and control policies and the adoption of formal prevention and control policies or lax prevention and control policies by local governments, resulting in periodic fluctuations of the epidemic^2^.

**Figure 1:**
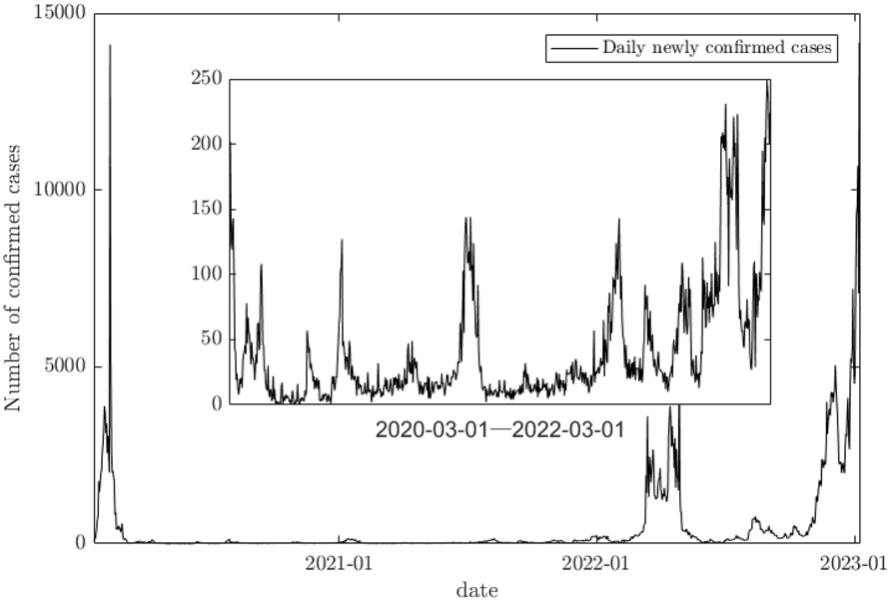
Daily confirmed cases in China from the COVID-19 outbreak to January 30, 2023 (Note: data from wind).

Figure 1 shows the daily new confirmed cases in China since the outbreak of COVID-19, which shows that the epidemic prevention and control policy adopted in China has achieved remarkable results, basically realizing "dynamic zero". However, small-scale outbreaks are cyclical after entering the normalized prevention and control stage. The daily new case data in Fig. 1 further reveal the fluctuation of the public and government collaborative prevention and control during the normalized prevention and control phase.

This outbreak is a classic public health emergency, and the prevention and control management of the epidemic will be affected by the cost of prevention and control, the benefit of prevention and control, and the enthusiasm of the public to cooperate^2^ etc. In the early epidemic stage, central government departments quickly formulated prevention and control policies, isolated and treated confirmed COVID-19 patients for free, established an epidemic tracing mechanism, and implemented "city closure. At the beginning of the epidemic, central government departments quickly formulated prevention policies, quarantined and treated confirmed COVID-19 patients free of charge, established an epidemic tracing mechanism, and implemented measures such as "city closure" and "road closure" to avoid further spread of the virus. At the same time, the public actively cooperated with the government in prevention and control for fear of contracting the new coronavirus. However, with repeated epidemic outbreaks, the public may be reluctant to actively cooperate with the government for prevention and control due to economic problems and the influence of foreign policies on epidemic prevention and control, such as choosing to move for livelihood^3^. Local governments may be lenient toward prevention and control due to economic development issues, social conflicts, and prevention and control costs^1^. Central government departments at this stage adhere to the principle of "scientific precision and dynamic At this stage, central government departments adhere to the principle of "scientific precision and dynamic zero," but they tend to intervene after an outbreak due to the randomness, lack of information, and multi-scope nature of the epidemic^4^.

Central government departments and local governments play a leading role in collaborative prevention and control, and the fluctuating problems in collaborative prevention and control are also closely related to the behaviors of government departments and the public. The reasons are: First, after the government increases the supervision and punishment of small-scale outbreaks due to the random flow of the public and the lax prevention and control of local governments, although this can reduce the random flow and lax prevention and control behaviors in the short term, it but can not achieve long-term governance effect, because once the government's supervision is lax, these behaviors will rebound. Secondly, excessive punishment by central government departments will also make the public and local governments more secretive and cautious when taking improper actions, thus making it more challenging to investigate and handle. It can be seen that the public and local governments' violations in collaborative prevention and control should not be punished more than once, and it is necessary to choose appropriate punishment strategies to control the stability of the system, which have rarely been explored in the existing literature.

Therefore, it is essential to theoretically explain the volatility of tripartite collaborative prevention and control, examine the specific interaction mechanism of strategic dependence of the public, local government, and central government departments, and explore the strategic choices of the game of the three subjects, and provide ideas and paths for volatility control to control the cyclical fluctuations of the epidemic.

## Review of relevant literature

The coordinated prevention and control of public health emergencies, as a systematic project, has not only received attention from government departments and the public, but also scholars at home and abroad have studied the causes of the problem of coordinated prevention and control fluctuations, related mathematical and rational model analysis, and response measures.

The public is the subject of prevention and control of public health emergencies, and the random movement of the public is an essential reason for the fluctuation and recurrence of epidemics. The intrinsic reason for this is the high cost of cooperation (cost of living, convenience of living) brought about by the social public's cooperation with government departments for prevention and control^3^, and thus the emergence of various types of undesirable behaviors of the social public that do not comply with prevention and control regulations^2^, including changing prevention and control behaviors out of self-interest^5^ and risk attitudes^6^. Government departments are the dominant players in epidemic prevention and control. Their different types and intensity of emergency management measures will have different guiding effects on the social public^4^. The effectiveness of collaborative prevention and control also depends on the level of preparation of local government departments^7^, with some local government departments generating a willingness to lax prevention and control due to regional economic development and intensification of social conflicts, resulting in small-scale epidemics that cannot be controlled in a timely and effective manner and large outbreaks. On the other hand, deficiencies in the regulatory system and emergency management system of central government departments are also one of the reasons for the repeated fluctuations of epidemics, such as over-reliance on extensive data regulation and lack of accountability^4^, over-emphasis on command and control and predictability^8^, and weak processes and applications of emergency management systems^9^. Therefore, our government should strengthen the investment in emergency management and improve the regulatory system to reduce the harm caused to the public and economy by public health emergencies^10,11^.

In addition, domestic and foreign scholars also analyze the epidemic through mathematical models, mainly through infectious disease dynamics models for scenario prediction, and their research results are more fruitful, including SIR models, SIS models, and SEIR models^12^. Shiva Moein^13^ found that SIR and its derivative models, which can only simulate the short-term spread of COVID-19, do not hold in the long-term predictions of the assumptions underlying SIR. This differs from the results predicted by Shaobo He^14^, who applied the SEIR model, who extrapolated the evolution of the epidemic in Hubei province considering the seasonal and stochastic nature of the epidemic, and showed that SEIR has better predictive properties for COVID-19. Similarly, the SIS model^15^ and SI model^16^ were applied to predict the infection rate of the epidemic. Second, the behavior of prevention and control subjects is analyzed by establishing game relationships through mathematical models, which mainly contain two aspects.

On the one hand, the models of centipede game^17^, cooperative game^18^, and sequential game^19^ are constructed based on the assumption of perfect rationality of classical game theory, such as literature^18^ to construct a cooperative game model between regions to explore the maximization of the overall regional cooperative effect under the minimization of the number of infections. On the other hand, it is based on finite rationality to construct evolutionary game and stochastic evolutionary game models to analyze the strategy choice of prevention and control subjects, such as Xiaochao Gui^20^ explored the evolutionary process of the stability of the mask switching and expansion coalition under COVID-19 by stochastic evolutionary game model; Fengjiao Chen and Dehai Liu^21^ applied evolutionary game to study the evolutionary path of multi-channel epidemic information dissemination model. In addition, literature^1,22,23^ applies the evolutionary game method to analyze the behavioral evolution path of each subject during the epidemic.

In addition to maintaining safe distances and wearing masks, government interventions often use reward and punishment mechanisms during epidemics and usually have sound motivational and deterrent effects^2^. The most commonly used reward and punishment mechanisms in various research fields are static reward and punishment mechanisms, which can be applied in the pharmaceutical field to regulate the production of inefficient drugs by pharmaceutical companies^24^, in the food field to provide positive incentives for food quality regulation^25^, and in the safety field^26^ to effectively avoid opportunistic behavior of private companies. Although static reward and punishment mechanisms accelerate the rate of system stabilization, they lose their usefulness for controlling fluctuating systems^27^. Therefore, dynamic reward and punishment mechanisms are gradually applied to control the stability of fluctuating systems; for example, the literature^28^ explores the effects of three reward and punishment mechanisms for green transformation of resource-based enterprises on the behavior of national ecological and environmental ministries, local ecological and environmental departments (bureaus), and resource-based enterprises. The study shows that nonlinear dynamic reward and punishment mechanisms have the best incentive effect.

Relevant scholars have made significant contributions to the study of public health emergencies and provided a specific basis for this paper. However, there are still three aspects that need to be improved: First, existing studies focus on analyzing the spread of epidemics and the effectiveness of measures from mathematical models of public health emergencies, with less research on the dynamic decision-making of different participants, especially the lack of considering the behavior of local governments and the public after the intervention of central government departments. Third, existing governmental reward and punishment mechanisms are usually static, and only the literature mentioned in the above review^28^ deals with dynamic reward and punishment mechanisms. At the same time, there needs to be more research on dynamic reward and punishment mechanisms in the control of volatility of public health emergency systems. In this paper, based on the existing studies, we propose that the study of dynamic reward and punishment mechanisms in the systemic volatility control of public health emergencies needs to be revised. Therefore, based on the existing research, this paper firstly reveals the volatility of the existing collaborative prevention and control through accurate data and then constructs a tripartite game model for the evolution of collaborative prevention and control based on the limited rational groups of the public, local government, and higher level government, visualizes the volatility of collaborative prevention and control through theoretical model explanation and data simulation, and finally introduces a dynamic punishment mechanism to control the volatility of the system. The game model is designed to provide theoretical support and a realistic basis for collaborative prevention and control among the public, local governments, and central government departments.

## Model assumptions and construction

The public health emergencies in this study involve three subjects: central government departments, local governments, and the public, and all of them are limited rational. The central government departments (e.g., the National Health and Welfare Commission) are not only the formulators of epidemic prevention and control strategies but also the regulators, adopting strategies such as (real-time regulation and periodic regulation). Real-time supervision means that the central government department formulates the epidemic prevention and control policy and then supervises the implementation of the policy by the local government and the flow of the public in real-time; periodic supervision means that the central government department formulates the policy and then leaves it to the local government department to implement it and the flow of the public, and then intervenes to control it after a public health emergency occurs. Local governments implement epidemic prevention and control policies and adopt strategies such as (strict prevention and control and lenient prevention and control). Strict prevention and control means that local governments actively implement prevention and control in accordance with the policy documents of the central government departments, such as during the COVID-19 outbreak, in accordance with the central government department's document "Notice on Strengthening Community Prevention and Control of Pneumonia Epidemic with Novel Coronavirus Infection "The implementation of grid and carpet management, "early detection, early reporting, early isolation, early diagnosis, early treatment", "dynamic zero" principle, etc.; lax prevention and control means that local governments did not follow the central government department's The lax prevention and control means that local governments do not actively carry out prevention and control according to the rules and regulations of the central government departments and do not pay sufficient attention to public health emergencies, such as the mitigation strategies adopted by the United States, India, and the United Kingdom in the face of the new crown pneumonia epidemic^12^. The public implements the epidemic prevention and control policy and adopts strategies such as (conscious isolation and casual mobility). Conscious isolation means that the public actively understands and cooperates with local epidemic prevention and control policies, such as actively reporting COVID-19-like symptoms such "fever, dry cough, and malaise", cooperating with prevention and control policies for conscious isolation and personal protection; casual mobility means that the public (confirmed infected persons, asymptomatic persons, close contacts, susceptible persons The public (confirmed infected persons, asymptomatic persons, close contacts, susceptible persons, etc.) disregard the epidemic prevention and control policy and move freely to and from various places only for their benefit.

### Model assumptions

#### Hypothesis 1

The probability that the public chooses the conscious isolation strategy is $x\in [0, 1]$ and the probability that chooses the discretionary flow strategy is $1-x$; the probability that the local government chooses the strict prevention and control strategy is $y\in [0, 1]$ and the probability that chooses the lax prevention and control strategy is $1-y$; the probability that the central government department chooses the real-time regulation is $z\in [0, 1]$ and the probability that chooses the periodic regulation is $1-z$.

#### Hypothesis 2

The social public actively cooperates with the epidemic prevention and control policy to isolate voluntarily will get the isolation subsidy $Q$ given by the local government, such as during COVID-19, the state issued the "Supplementary Notice on the Medical Protection Work of Pneumonia Epidemic Infected by New Coronavirus" that the part of the medical expenses of the new coronavirus patients or suspected patients with personal burden will be subsidized by the local government, but they need to pay the time cost of isolation ${C}_{1}$. If the public ignores the prevention and control policy to flow at will, still meeting friends, office, travel to obtain the benefits of $V$, but if the public due to the random flow of the epidemic spread, in addition to pay for personal inspection, protection costs $E$, there will be the cost of punishment when the local government strict prevention and control $R$. For example, China provides that individuals who violate the provisions of the epidemic private flow, concealment, false reporting constitutes the spread of the epidemic, suspected of endangering public security crime, which is punished by the public security department^29^; when the local government is lax in prevention and control, it cannot fully grasp the flow of the social public, but has a certain probability of discovering the random flow of the social public, and this probability is noted as $P$. The cost of punishment of the social public under the lax prevention and control of the local government is $PR$, and $P$ on the one hand indicates the probability of discovering the random flow of the social public when the local government is lax in prevention and control. On the other hand, it portrays the degree of lax prevention and control of the local government; if the social public is consciously segregated and the casual flow is supervised by the central government department in real time, there will be reward ${M}_{1}$ and punishment ${N}_{1}$ respectively, and the reward includes issuing consumption vouchers, free medical checkups or other economic rewards, and the punishment includes fines or other non-economic penalties.

#### Hypothesis 3

The benefit to the local government when the public is voluntarily isolated is $H$, and the burden to the local government when it is randomly mobile is $S$. When the local government is strict in prevention and control, the cost of prevention and control is $C$, and the cost of lax prevention and control is $kC$, $k\in [0, 1]$, and the measures for strict prevention and control include timely disinfection of public places, implementation of the "collect as much as possible" policy for infected people The measures of strict prevention and control include timely disinfection of public places, implementation of the policy of "collecting as many infected people as possible", controlling the source of infection, and cutting off the transmission route^12^. If the local government's strict prevention and control and lax prevention and control are monitored by the central government department in real time, there will be reward ${M}_{2}$ and penalty ${N}_{2}$.

#### Hypothesis 4

The central government department adopts a real-time regulatory strategy and needs to pay regulatory costs ${V}_{1}$, including labor costs and time costs for regulation. If the public consciously quarantine and the local government strictly prevent and control, the central government department will gain benefits $T$ at this time, including stable social and economic development, etc. If the local government chooses lax prevention and control and the public chooses to move freely, resulting in the widespread spread of the epidemic, the central government department will incur economic losses recorded as $D$. Such losses include financial support from the central government department and mobilization of medical personnel to support the epidemic site. If the central government department adopts a cyclical control strategy, it cannot obtain real-time information about the behavior of the public and local governments.

### Model construction

Based on the above research hypotheses, this paper constructs a game payment matrix with mixed strategies for the public, local government and central government departments, as shown in Table 1.

**Table 1:** Mixed strategy game payment matrix for the public, local government, and central government departments.

Portfolio of gaming strategies	Social public benefits	Local government revenue	Central government sector earnings
① (conscious isolation, strict prevention and control, real-time supervision)	$Q+{M}_{1}-{C}_{1}$	$H+{M}_{2}-C-Q$	$T-{M}_{1}-{M}_{2}-{V}_{1}$
② (conscious isolation, strict prevention and control, cycle regulation)	$Q-{C}_{1}$	$H-C-Q$	$T$
③ (conscious isolation, lenient prevention and control, real-time supervision)	$Q+{M}_{1}-{C}_{1}$	$H-kC-Q-{N}_{2}$	${N}_{2}-{M}_{1}-{V}_{1}$
④ (conscious isolation, lenient prevention and control, cycle regulation)	$Q-{C}_{1}$	$H-kC-Q$	$0$
⑤ (casual flow, strict prevention and control, real-time supervision)	$V-R-{N}_{1}-E$	$R+{M}_{2}-C-S$	${N}_{1}-{M}_{2}-{V}_{1}$
⑥ (casual flow, strict prevention and control, cycle regulation)	$V-R-E$	$R-C-S$	$0$
⑦ (casual flow, lenient prevention and control, real-time supervision)	$V-PR-E-{N}_{1}$	$PR-kC-{N}_{2}-S$	${N}_{1}+{N}_{2}-D-{V}_{1}$
⑧ (casual flow, lenient prevention and control, cycle regulation)	$V-PR-E$	$PR-kC-S$	$-D$

The payment matrix of the mixed strategy game in Table 1 gives:

The expected benefit for the public to choose the "conscious segregation" strategy is ${E}_{A1}$ and the expression is:

1 $${E}_{A1}=yz\left(Q+{M}_{1}-{C}_{1}\right)+y\left(1-z\right)\left(Q-{C}_{1}\right)+\left(1-y\right)z\left(Q+{M}_{1}-{C}_{1}\right)+\left(1-y\right)\left(1-z\right)\left(Q-{C}_{1}\right)$$

The expected return when choosing the "discretionary flow" strategy is ${E}_{A2}$ and the expression is:

2 $${E}_{A2}=yz\left(V-R-{N}_{1}-E\right)+y\left(1-z\right)\left(V-R-E\right)+\left(1-y\right)z\left(V-PR-E-{N}_{1}\right)+\left(1-y\right)\left(1-z\right)\left(V-PR-E\right)$$

The average expected return of the public is $\overline{{E }_{A}}$, The expression is:

3 $$\overline{{E }_{A}}=x{E}_{A1}+\left(1-x\right){E}_{A2}$$

The replication dynamic equation for the public's choice of the "conscious isolation" strategy can be obtained as follows:

4 $$F\left(x\right)=\frac{dx}{dt}=x\left({E}_{A1}-\overline{{E }_{A}}\right) =x\left(1-x\right)\left[E+Q+PR-V-{C}_{1}+\left({M}_{1}+{N}_{1}\right)z+\left(R-PR\right)y\right]$$

Similarly, the replication dynamic equations for the "strict prevention and control" strategy chosen by the local government and the "real-time supervision" strategy chosen by the central government are shown in Eqs. (5) and (6), respectively.

5 $$F\left(y\right)=\frac{dy}{dt}=y\left({E}_{B1}-\overline{{E }_{B}}\right) =y\left(1-y\right)\left[R+kC-C-PR+\left({M}_{2}+{N}_{2}\right)z+\left(PR-R\right)x\right]$$

6 $$F\left(z\right)=\frac{dz}{dt}=z\left({E}_{C1}-\overline{{E }_{C}}\right) =z\left(1-z\right)\left[{N}_{1}+{N}_{2}-{V}_{1}-\left({M}_{1}+{N}_{1}\right)x-\left({M}_{2}+{N}_{2}\right)y\right]$$

where ${E}_{B1}$ represents the expected return for local governments choosing the "strict prevention and control" strategy and ${E}_{C1}$ represents the expected return for central government departments choosing the "real-time regulation" strategy.

## Model analysis

### Stabilization strategy of the probability of the public choosing the "conscious isolation" strategy

The probability of the public choosing the "conscious isolation" strategy is $x$. The replicated dynamic equation is:

$$F\left(x\right)=\frac{dx}{dt}=x\left({E}_{A1}-\overline{{E }_{A}}\right)$$

Taking the derivative of $F(x)$ with respect to $x$ yields:

7 $$\frac{d(F\left(x\right))}{dx}=\left(1-2x\right)\left[E+Q+PR-V-{C}_{1}+\left({M}_{1}+{N}_{1}\right)z+\left(R-PR\right)y\right]$$

Set

8 $$G\left(y, z\right)=E+Q+PR-V-{C}_{1}+\left({M}_{1}+{N}_{1}\right)z+\left(R-PR\right)y$$

From $G(y, z)=0$, it follows that:

When $y=\frac{V+{C}_{1}-E-Q-PR-\left({M}_{1}+{N}_{1}\right)z}{R-PR}$, $\frac{d(F\left(x\right))}{dx}\equiv 0$; when $y\ne \frac{V+{C}_{1}-E-Q-PR-\left({M}_{1}+{N}_{1}\right)z}{R-PR}$, let $F(x)=0$, then $x=0$ and $x=1$ are two equilibrium points, so they need to be discussed categorically.

#### Corollary 1

* If the probability of local government choosing the "strict prevention and control" strategy is higher than* $\frac{V+{C}_{1}-E-Q-PR-\left({M}_{1}+{N}_{1}\right)z}{R-PR}$,* then the probability of the public choosing the "conscious isolation" strategy will be stable at 1. Conversely, if the probability of local government choosing the "strict prevention and control" strategy is lower than* $\frac{V+{C}_{1}-E-Q-PR-\left({M}_{1}+{N}_{1}\right)z}{R-PR}$,* then the probability of the public choosing the "conscious isolation" strategy will be stable at 0*.

#### Proof

$\frac{d(G\left(y, z\right))}{dy}=R\left(1-P\right)>0$ so $G\left(y, z\right)$ is an increasing function about y. When $y>\frac{V+{C}_{1}-E-Q-PR-\left({M}_{1}+{N}_{1}\right)z}{R-PR}$, $G\left(y, z\right)>0$, so we have $\frac{d(F\left(x\right))}{dx}{|}_{x=0}>0$, $\frac{d(F\left(x\right))}{dx}{|}_{x=1}<0$; when $y<\frac{V+{C}_{1}-E-Q-PR-\left({M}_{1}+{N}_{1}\right)z}{R-PR}$,$G\left(y, z\right)<0$, so we have $\frac{d(F\left(x\right))}{dx}{|}_{x=0}<0$ and $\frac{d(F\left(x\right))}{dx}{|}_{x=1}>0$.

In summary, the response function for the probability $x$ of the public choosing the "conscious isolation" strategy is

9 $$x=\left\{\begin{array}{l}0 if y<\frac{V+{C}_{1}-E-Q-PR-\left({M}_{1}+{N}_{1}\right)z}{R-PR}\\ \left[0,1\right] if y=\frac{V+{C}_{1}-E-Q-PR-\left({M}_{1}+{N}_{1}\right)z}{R-PR}\\ 1 if y>\frac{V+{C}_{1}-E-Q-PR-\left({M}_{1}+{N}_{1}\right)z}{R-PR}\end{array}\right.$$

**Figure 2:**
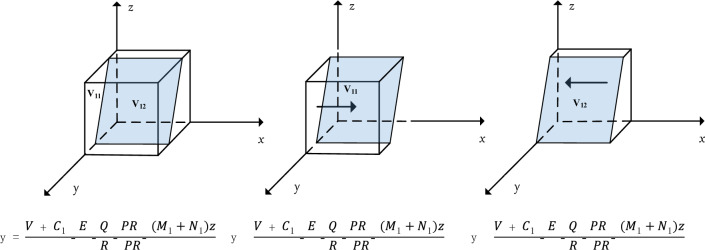
Trend of evolutionary dynamics of social public.

At this time, the replication dynamics and evolutionary stabilization strategy of the public's choice of "conscious isolation" strategy are shown in Fig. 2.

Corollary 1 suggests that in the prevention and control of public health emergencies if local governments tend to choose strict prevention and control, the public tends to choose conscious isolation, while whether the central government department chooses real-time or periodic regulation, it has little impact on local government gains; if the public chooses casual mobility, it will be subject to higher penalties. Conversely, if the local government chooses lax regulation, then the social public will choose casual mobility.

It can be seen by Fig. 2 that $y=\frac{V+{C}_{1}-E-Q-PR-\left({M}_{1}+{N}_{1}\right)z}{R-PR}$ divides $\Omega =\{N(x,y,z)|0\le x\le \mathrm{1,0}\le y\le \mathrm{1,0}\le z\le 1\}$ into two spaces ${V}_{11}$ and ${V}_{12}$, ${V}_{11}$ represents the probability of the social public choosing conscious segregation and ${V}_{12}$ represents the probability of the social public choosing casual mobility. It is calculated that.

$${V}_{12}={\int }_{0}^{1}{\int }_{0}^{1}\frac{V+{C}_{1}-E-Q-PR-\left({M}_{1}+{N}_{1}\right)z}{R-PR}dzdx$$

10 $$=\frac{2V+2{C}_{1}-2E-2Q-2PR-{M}_{1}-{N}_{1}}{2(R-PR)}$$

$${V}_{11}=1-{\int }_{0}^{1}{\int }_{0}^{1}\frac{V+{C}_{1}-E-Q-PR-\left({M}_{1}+{N}_{1}\right)z}{R-PR}dzdx$$

11 $$=1-\frac{2V+2{C}_{1}-2E-2Q-2PR-{M}_{1}-{N}_{1}}{2(R-PR)}$$

#### Corollary 2

*The subsidy* $Q$* given by the local government to consciously segregate the social public, the probability* $P$* and penalty* $R$* of the local government finding the social public to move freely, the cost* $E$* paid by the individual when the social public is found to move freely, the penalty* ${N}_{1}$* given by the central government department finding the social public to move freely, and the reward* ${M}_{1}$* given by the central government department finding the social public to move freely have a positive incentive effect on the social public choosing the "consciously segregated" strategy; the benefit* $V$* of the social public moving freely and the time cost* ${C}_{1}$* of consciously segregating have a negative incentive effect on the social public choosing the "consciously segregated" strategy*.

#### Proof

According to Eq. (10), $0<2V+2{C}_{1}-2E-2Q-2PR-{M}_{1}-{N}_{1}<2(R-PR)$, the probability $x$ of the public choosing the "voluntary segregation" strategy, i.e., ${V}_{11}$, is obtained by taking the partial derivatives of $V, {C}_{1}, E, Q, P, R, {M}_{1}$, and ${N}_{1}$. It is obtained that:

12 $$\left\{\begin{array}{c}\frac{\partial ({V}_{11})}{\partial V}=\frac{\partial ({V}_{11})}{\partial {C}_{1}}=-\frac{1}{R-PR}<0\\ \frac{\partial ({V}_{11})}{\partial E}=\frac{\partial ({V}_{11})}{\partial Q}=\frac{1}{R-PR}>0\\ \frac{\partial ({V}_{11})}{\partial {M}_{1}}=\frac{\partial ({V}_{11})}{\partial {N}_{1}}=\frac{1}{2(R-PR)}>0\\ \frac{\partial ({V}_{11})}{\partial R}=\frac{2P\left(2R-2PR\right)+2(2V+2{C}_{1}-2E-2Q-2PR-{M}_{1}-{N}_{1})}{2{(R-PR)}^{2}}>0\\ \frac{\partial ({V}_{11})}{\partial P}=\frac{2\left(R-PR\right)-(2V+2{C}_{1}-2E-2Q-2PR-{M}_{1}-{N}_{1})}{2(1-P)(R-PR)}>0\end{array}\right.$$

Therefore, ${V}_{11}$ is an increasing function with respect to $E, Q, {M}_{1}, {N}_{1}, R$, and $P$. That is, as $E, Q, {M}_{1}, {N}_{1}, R$ and $P$ increase, ${V}_{11}$ gradually increases, and the probability of the public choosing the "conscious isolation" strategy increases; as $V, {C}_{1}$ decreases, ${V}_{11}$ gradually decreases.

Corollary 2 suggests that when the cost of choosing discretionary mobility is high, choosing the "discretionary mobility" strategy is not worth the cost, and the public tends to choose the "conscious isolation" strategy.

### Local governments choose "strict prevention and control" strategy probability of stabilization strategy

The probability that the local government chooses the "strict prevention and control" strategy is $y$. The replicated dynamic equation is

$$F\left(y\right)=\frac{dy}{dt}=y\left({E}_{B1}-\overline{{E }_{B}}\right)$$

Taking the derivative of F(y) with respect to $y$ gives:

13 $$\frac{d(F\left(y\right))}{dy}=\left(1-2y\right)\left[R+kC-C-PR+\left({M}_{2}+{N}_{2}\right)z+\left(PR-R\right)x\right]$$

Set

14 $$H\left(x, z\right)=R+kC-C-PR+\left({M}_{2}+{N}_{2}\right)z+\left(PR-R\right)x$$

From $H\left(x, z\right)=0$, it follows that:

When $x=\frac{R+kC-C-PR+\left({M}_{2}+{N}_{2}\right)z}{R-PR}$, $F(y)\equiv 0$; when $x\ne \frac{R+kC-C-PR+\left({M}_{2}+{N}_{2}\right)z}{R-PR}$, let $F\left(y\right)=0$. Then $y=0$ and $y=1$ are two equilibrium points, so it needs to be discussed categorically.

#### Corollary 3

*If the probability of the public choosing the "conscious isolation" strategy is lower than* $\frac{R+kC-C-PR+\left({M}_{2}+{N}_{2}\right)z}{R-PR}$,* then the probability of the local government choosing the "strict prevention and control" strategy will be stable at 1. Conversely, if the probability of the public choosing the "conscious isolation" strategy is higher than* $\frac{R+kC-C-PR+\left({M}_{2}+{N}_{2}\right)z}{R-PR}$,* then the probability of the local government choosing the "strict prevention and control" strategy will be stable at 0*.

#### Proof

$\frac{dH\left(x, z\right))}{dx}=PR-R<0$,so $H(x, z)$ is a decreasing function about x. When $x>\frac{R+kC-C-PR+\left({M}_{2}+{N}_{2}\right)z}{R-PR}$, $H\left(x, z\right)<0$, so we have $\frac{d(F\left(y\right))}{dy}{|}_{y=0}<0$ and $\frac{d(F\left(y\right))}{dy}{|}_{y=1}>0$.When $x<\frac{R+kC-C-PR+\left({M}_{2}+{N}_{2}\right)z}{R-PR}$, $H\left(x, z\right)>0$, so we have $\frac{d(F\left(y\right))}{dy}{|}_{y=0}>0$ and $\frac{d(F\left(y\right))}{dy}{|}_{y=1}<0$.

At this point, the response function of the probability y that the local government chooses the "strict prevention and control" strategy is.

15 $$y=\left\{\begin{array}{l}0 \quad \quad if x>\frac{R+kC-C-PR+\left({M}_{2}+{N}_{2}\right)z}{R-PR}\\ \left[0,1\right]\quad \quad if x=\frac{R+kC-C-PR+\left({M}_{2}+{N}_{2}\right)z}{R-PR}\\ 1\quad \quad if x<\frac{R+kC-C-PR+\left({M}_{2}+{N}_{2}\right)z}{R-PR}\end{array}\right.$$

**Figure 3:**
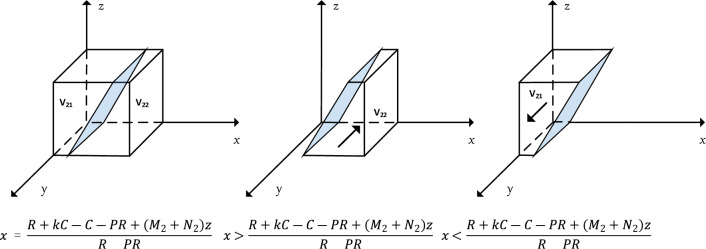
Trends in the evolutionary dynamics of local governments.

The replication dynamics and evolutionary stabilization strategies of local governments choosing the "strict prevention and control" strategy are shown in Fig. 3.

Corollary 3 suggests that in the prevention and control of public health emergencies, if the public is more conscious, aware of safety and self-protection, and tends to adopt a conscious isolation strategy, then the local government will choose a "lenient prevention and control" strategy in consideration of maximizing its interests; conversely, if the public is less conscious and tends to adopt a random flow strategy, then the local government will eventually choose a "strict prevention and control" strategy to deal with public health emergencies.

It can be seen by Fig. 3 that $x=\frac{R+kC-C-PR+\left({M}_{2}+{N}_{2}\right)z}{R-PR}$ divides $\Omega =\{N(x,y,z)|0\le x\le \mathrm{1,0}\le y\le \mathrm{1,0}\le z\le 1\}$ into two spaces ${V}_{21}$ and ${V}_{22}$, ${V}_{21}$ represents the probability of local government choosing strict prevention and control, and ${V}_{22}$ represents the probability of local government choosing lax prevention and control. The calculation yields.

16 $${V}_{21}={\int }_{0}^{1}{\int }_{0}^{1}\frac{R+kC-C-PR+\left({M}_{2}+{N}_{2}\right)z}{R-PR}dzdy=\frac{2\left(R+kC-C-PR\right)+{M}_{2}+{N}_{2}}{2(R-PR)}$$

#### Corollary 4

The penalty $R$ for local government to find out the free flow of social public, the cost saving coefficient $k$ for local government's lax regulation, the reward ${M}_{2}$ for central government department to find out local government's strict prevention and control, and the penalty ${N}_{2}$ for central government department to find out local government's lax prevention and control have positive incentive effects on local government's choice of "strict prevention and control" strategy. The probability $P$ that the local government finds that the public is free to move and the cost $C$ that the local government's strict prevention and control policies negatively incentivize the local government's choice of "strict prevention and control."

#### Proof

The probability y that the local government chooses the "strict prevention and control" strategy, i.e., ${V}_{21}$, is partial derivative with respect to $R, k, C, P, {M}_{2}, {N}_{2}$, and the derivative is referred to Eq. (12), which is omitted here.

The results show that ${V}_{21}$ is an increasing function with respect to $R, k, {M}_{2}$, and ${N}_{2}$. As $R, k, {M}_{2}$, and ${N}_{2}$ increase, ${V}_{21}$ gradually increases, and the probability of local governments choosing "strict prevention and control" strategy increases; as $P$ and $C$ decrease, ${V}_{21}$ gradually decreases.

Corollary 4 suggests that the incentive and punishment mechanism of central government departments to local governments is essential for local governments to adopt a "strict prevention and control" strategy. When the cost of strict prevention and control is high, local governments will be less willing to choose strict prevention and control based on profit maximization; when local governments choose a lax regulatory strategy to cause large-scale local epidemic spread or when the expected benefit of strict prevention and control is high, local government departments will increase the probability of strict prevention and control.

### Stabilization strategy for the probability of choosing a "real-time regulation" strategy for central government departments

The probability of a central government department choosing the "real-time regulation" strategy is $z$. The replication dynamic equation is

$$F\left(z\right)=\frac{dz}{dt}=z\left({E}_{C1}-\overline{{E }_{C}}\right)$$

Taking the derivative of $F(z)$ with respect to $z$ yields

17 $$\frac{d(F\left(z\right))}{dz}=\left(1-2z\right)\left[{N}_{1}+{N}_{2}-{V}_{1}-\left({M}_{1}+{N}_{1}\right)x-\left({M}_{2}+{N}_{2}\right)y\right]$$

Set

18 $$K\left(x, y\right)={N}_{1}+{N}_{2}-{V}_{1}-\left({M}_{1}+{N}_{1}\right)x-\left({M}_{2}+{N}_{2}\right)y$$

From $K\left(x, y\right)=0$, it follows that

When $x=\frac{{N}_{1}+{N}_{2}-{V}_{1}-\left({M}_{2}+{N}_{2}\right)y}{{M}_{1}+{N}_{1}}$, $F(z)\equiv 0$; when $x\ne \frac{{N}_{1}+{N}_{2}-{V}_{1}-\left({M}_{2}+{N}_{2}\right)y}{{M}_{1}+{N}_{1}}$, let $F(z)=0$. Then $z=0$ and $z=1$ are two equilibrium points, so it needs to be discussed categorically.

#### Corollary 5

*If the probability of the public choosing the "conscious segregation" strategy is lower than* $\frac{{N}_{1}+{N}_{2}-{V}_{1}-\left({M}_{2}+{N}_{2}\right)y}{{M}_{1}+{N}_{1}}$,* then the probability of the central government choosing the "real-time regulation" strategy will be stable at 1. Conversely, if the probability of the public choosing the "conscious segregation" strategy is higher than* $\frac{{N}_{1}+{N}_{2}-{V}_{1}-\left({M}_{2}+{N}_{2}\right)y}{{M}_{1}+{N}_{1}}$,* then the probability of the central government choosing the "real-time regulation" strategy will be stable at 0*.

#### Proof

$\frac{dK\left(x, y\right)}{dx}=-\left({M}_{1}+{N}_{1}\right)<0$, so $K\left(x, y\right)$ is a decreasing function about x. When $x>\frac{{N}_{1}+{N}_{2}-{V}_{1}-\left({M}_{2}+{N}_{2}\right)y}{{M}_{1}+{N}_{1}}$, $K\left(x, y\right)<0$, so we have $\frac{d(F\left(z\right))}{dz}{|}_{z=0}<0$, $\frac{d(F\left(z\right))}{dz}{|}_{z=1}>0$; when $x<\frac{{N}_{1}+{N}_{2}-{V}_{1}-\left({M}_{2}+{N}_{2}\right)y}{{M}_{1}+{N}_{1}}$, $K\left(x, y\right)>0$, so we have $\frac{d(F\left(z\right))}{dz}{|}_{z=0}>0$ and $\frac{d(F\left(z\right))}{dz}{|}_{z=1}<0$.

At this point, the response function for the probability z of the central government department choosing the "real-time regulation" strategy is:

19 $$z=\left\{\begin{array}{l}0 \quad \quad if x>\frac{{N}_{1}+{N}_{2}-{V}_{1}-\left({M}_{2}+{N}_{2}\right)y}{{M}_{1}+{N}_{1}}\\ \left[0,1\right]\quad \quad if x=\frac{{N}_{1}+{N}_{2}-{V}_{1}-\left({M}_{2}+{N}_{2}\right)y}{{M}_{1}+{N}_{1}}\\ 1\quad \quad if x<\frac{{N}_{1}+{N}_{2}-{V}_{1}-\left({M}_{2}+{N}_{2}\right)y}{{M}_{1}+{N}_{1}}\end{array}\right.$$

**Figure 4:**
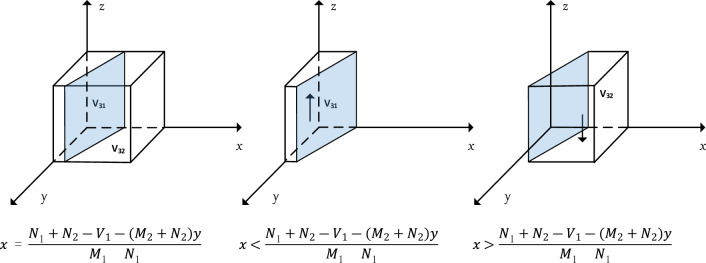
Trend of evolutionary dynamics of central government departments.

At this point, the central government department chooses the "real-time regulation" strategy of replication dynamics and evolutionary stability strategy as shown in Fig. 4.

It can be seen through Fig. 4 that $x=\frac{{N}_{1}+{N}_{2}-{V}_{1}-\left({M}_{2}+{N}_{2}\right)y}{{M}_{1}+{N}_{1}}$ divides $\Omega =\{N(x,y,z)|0\le x\le \mathrm{1,0}\le y\le \mathrm{1,0}\le z\le 1\}$ into two spaces ${V}_{31}$ and ${V}_{32}$, with ${V}_{31}$ representing the probability that the central government department chooses real-time regulation and ${V}_{32}$ representing the probability that the central government department chooses periodic probability of regulation. The calculation yields.

20 $${\int }_{0}^{1}{\int }_{0}^{1}\frac{{N}_{1}+{N}_{2}-{V}_{1}-\left({M}_{2}+{N}_{2}\right)y}{{M}_{1}+{N}_{1}}dydz=\frac{{N}_{1}-{V}_{1}-{M}_{2}}{{M}_{1}+{N}_{1}}$$

#### Corollary 6

*The penalty* ${N}_{1}$* for the central government department to discover the free flow of the public has a positive incentive effect on the central government department to choose the "real-time regulation" strategy; the cost* ${V}_{1}$* for the central government department to regulate in real time, and the incentives* ${M}_{1}$* and* ${M}_{2}$* for the central government department to voluntarily isolate the public and strictly prevent and control the local government have a negative incentive effect on the central government department to choose the "real-time regulation" strategy*.

#### Proof

The probability $z$ that the central government department chooses the "real-time regulation" strategy, i.e., ${V}_{31}$, is biased with respect to ${M}_{1}$, ${N}_{1}$, ${M}_{2}$, and ${V}_{1}$, and the derivative is referred to Eq. (12), which is omitted here.

The results show that ${V}_{31}$ is an increasing function about ${N}_{1}$, and as ${N}_{1}$ keeps increasing, ${V}_{31}$ gradually increases and the probability of central government departments adopting real-time regulatory strategies keeps increasing; ${V}_{31}$ is a decreasing function about ${M}_{1}, {M}_{2}$ , and ${V}_{1}$, and as ${M}_{1}, {M}_{2}$ , and ${V}_{1}$ increase, ${V}_{31}$ gradually decreases and the probability of central government departments adopting real-time regulatory strategies keeps decreasing.

Corollary 6 suggests that the penalties imposed on the public when the central government department regulates in real-time will motivate the central government department to adopt the real-time regulation strategy. However, the willingness to adopt the real-time regulation strategy will be reduced when the cost of real-time regulation is high, and the rewards for compliance by local governments and the public should be lowered.

### Evolutionary stability analysis of the system

From Eqs. (4), (5) and (6), the set of replicated dynamic equations is formed, and making this set of equations 0 yields eight equilibrium points of the replicated dynamic system, namely ${E}_{1}(\mathrm{0,0},0), {E}_{2}(\mathrm{0,1},0), {E}_{3}(\mathrm{0,0},1), {E}_{4}(\mathrm{0,1},1), {E}_{5}(\mathrm{1,0},0), {E}_{6}$(1,1,0), ${E}_{7}$(1,0,1), ${E}_{8}$(1,1,1), where the stability of the hybrid equilibrium point of the replicated dynamical system is not considered, since the hybrid equilibrium point must not be ESS in the asymmetric evolutionary game.

According to Lyapunov's theorem, the stability of the replicated dynamic system can be judged by the eigenvalues of the Jacobi matrix composed of the set of replicated dynamic equations. When the fundamental part of the eigenvalues of the Jacobi matrix are all negative, the equilibrium has asymptotic stability, and if at least one of the eigenvalues is not harmful, the equilibrium is unstable; after that, the Jacobi is expressed as J:

$$J=\left[\begin{array}{cc}\left(1-2x\right)\left[E+Q+PR-V-{C}_{1}+\left({M}_{1}+{N}_{1}\right)z+\left(R-PR\right)y\right]& \begin{array}{cc}x\left(1-x\right)\left(R-PR\right)& x\left(1-x\right)\left({M}_{1}+{N}_{1}\right)\end{array}\\ \begin{array}{c}y\left(1-y\right)\left(PR-R\right)\\ -z\left(1-z\right)\left({M}_{1}+{N}_{1}\right)\end{array}& \begin{array}{c}\begin{array}{cc}\left(1-2y\right)\left[R+kC-C-PR+\left({M}_{2}+{N}_{2}\right)z+\left(PR-R\right)x\right]& y\left(1-y\right)\left({M}_{2}+{N}_{2}\right)\end{array}\\ \begin{array}{cc}-z\left(1-z\right)\left({M}_{2}+{N}_{2}\right)& \left(1-2z\right)\left[{N}_{1}+{N}_{2}-{V}_{1}-\left({M}_{1}+{N}_{1}\right)x-\left({M}_{2}+{N}_{2}\right)y\right]\end{array}\end{array}\end{array}\right]$$

Bringing ${E}_{1}\sim {E}_{8}$ into the Jacobi matrix, the eigenvalues and stability of the equilibrium points can be found as shown in Table 2.

**Table 2:** Equilibrium point stability analysis table.

Balancing point	${\lambda }_{1}$	${\lambda }_{2}$	${\lambda }_{3}$	Symbols	Stability conclusion	ESS determination conditions
${E}_{1}$(0,0,0)	$E-{C}_{1}+Q-V+PR$	$R-C-PR+kC$	${N}_{1}+{N}_{2}-{V}_{1}$	$(\times ,\times ,\times )$	Instability point or ESS	①
${E}_{2}$(0,1,0)	$E-{C}_{1}+Q+R-V$	$C-R+PR-kC$	${N}_{1}-{M}_{2}-{V}_{1}$	$(\times ,\times ,\times )$	Instability point or ESS	②
${E}_{3}$(0,0,1)	$E-{C}_{1}+{M}_{1}+{N}_{1}+Q-V+PR$	${M}_{2}+{N}_{2}-C+R-PR+kC$	${V}_{1}-{N}_{1}-{N}_{2}$	$(\times ,\times ,\times )$	Instability point or ESS	③
${E}_{4}$(0,1,1)	$E-{C}_{1}+{M}_{1}+{N}_{1}+Q+R-V$	$C-{M}_{2}-{N}_{2}-R+PR-kC$	${M}_{2}-{N}_{1}+{V}_{1}$	$(\times ,\times ,\times )$	Instability point or ESS	④
${E}_{5}$(1,0,0)	${C}_{1}-E-Q+V-PR$	$kC-C$	${N}_{2}-{M}_{1}-{V}_{1}$	$(\times ,-,\times )$	Instability point or ESS	⑤
${E}_{6}$(1,1,0)	${C}_{1}-E-Q+V-R$	$C-kC$	$-{M}_{1}-{M}_{2}-{V}_{1}$	$(\times ,+,-)$	Instability point	/
${E}_{7}$(1,0,1)	${C}_{1}-E-{M}_{1}-{N}_{1}-Q+V-PR$	${M}_{2}+{N}_{2}+kC-C$	${M}_{1}-{N}_{2}+{V}_{1}$	$(\times ,\times ,\times )$	Instability point or ESS	⑥
${E}_{8}$(1,1,1)	${C}_{1}-E-{M}_{1}-{N}_{1}-Q+V-R$	$C-{M}_{2}-{N}_{2}-kC$	${M}_{1}+{M}_{2}+{V}_{1}$	$(\times ,\times ,+)$	Instability point	/

× indicates that the sign is uncertain, ∕ indicates the presence of non-negative eigenvalues.① $E-{C}_{1}+Q-V+PR<0$, $R-C-PR+kC<0$, ${N}_{1}+{N}_{2}-{V}_{1}<0$; ② $E-{C}_{1}+Q+R-V<0$, $C-R+PR-kC<0$, ${N}_{1}-{M}_{2}-{V}_{1}<0$; ③ $E-{C}_{1}+{M}_{1}+{N}_{1}+Q-V+PR<0$, ${M}_{2}+{N}_{2}-C+R-PR+kC<0$, ${V}_{1}-{N}_{1}-{N}_{2}<0$; ④ $E-{C}_{1}+{M}_{1}+{N}_{1}+Q+R-V<0$, $C-{M}_{2}-{N}_{2}-R+PR-kC<0$, ${M}_{2}-{N}_{1}+{V}_{1}<0$; ⑤ ${C}_{1}-E-Q+V-PR<0$, ${N}_{2}-{M}_{1}-{V}_{1}<0$; ⑥ ${C}_{1}-E-{M}_{1}-{N}_{1}-Q+V-PR<0$, ${M}_{2}+{N}_{2}+kC-C<0$, ${M}_{1}-{N}_{2}+{V}_{1}<0.$

From Table 2, the stable points of the system are ${E}_{1}(\mathrm{0,0},0), {E}_{2}(\mathrm{0,1},0), {E}_{3}(\mathrm{0,0},1), {E}_{4}(\mathrm{0,1},1), {E}_{5}(\mathrm{1,0},0), {E}_{7}$(1,0,1) and ${E}_{1}$, ${E}_{2}$, ${E}_{3}$, and ${E}_{4}$ equilibrium points in which the social public evolves to the random flow strategy. should increase the subsidy for the social public's conscious isolation, the cost of personal protection for the social public and the cost of punishment for the social public's random flow, and the reward for conscious isolation, at least so that $E+Q+PR<{V+C}_{1}$; and for equilibrium points ${E}_{5}$ and ${E}_{7}$, the social public eventually evolves to conscious isolation, but the local government eventually evolves to lax prevention and control, and the central government department can achieve the strict prevention and control of the local government through rewards and punishments in real-time supervision. However, this equilibrium is not the most ideal strategy for evolutionary stability. In fact, from the perspective of managers, ${E}_{6}$(1,1,0) is a Pareto optimal solution, but it is difficult to achieve the pure strategy equilibrium of ${E}_{6}$ under the static reward and punishment mechanism, so this paper will further consider the dynamic reward and punishment mechanism to achieve the mixed strategy equilibrium of ${E}_{6}$.

## Numerical simulation

In order to visually reveal the interaction of the behaviors of the three subjects, Matlab 2016b is used to visualize the behaviors of the public and local government. Central government departments and the effects of initial willingness on the evolutionary results and the effects of parameter changes on the evolutionary results are considered separately, and stability control strategies are provided. The initial values of the relevant parameters are set by a certain proportion according to the COVID-19 period, such as the isolation allowance standard of 300/day for 14 days in Tianjin, and the remaining parameter settings are specified in Table 3.

**Table 3:** Initial parameters.

Parameters	$E$	$Q$	$P$	$R$	$V$	${C}_{1}$	${M}_{1}$	${N}_{1}$	$k$	$C$	${M}_{2}$	${N}_{2}$	${V}_{1}$
Numerical value	0.6	4.2	0.8	0.5	5.6	2.8	5	1.6	0.2	8	5	10	4

### Numerical simulation of single-party body stability

**Figure 5:**
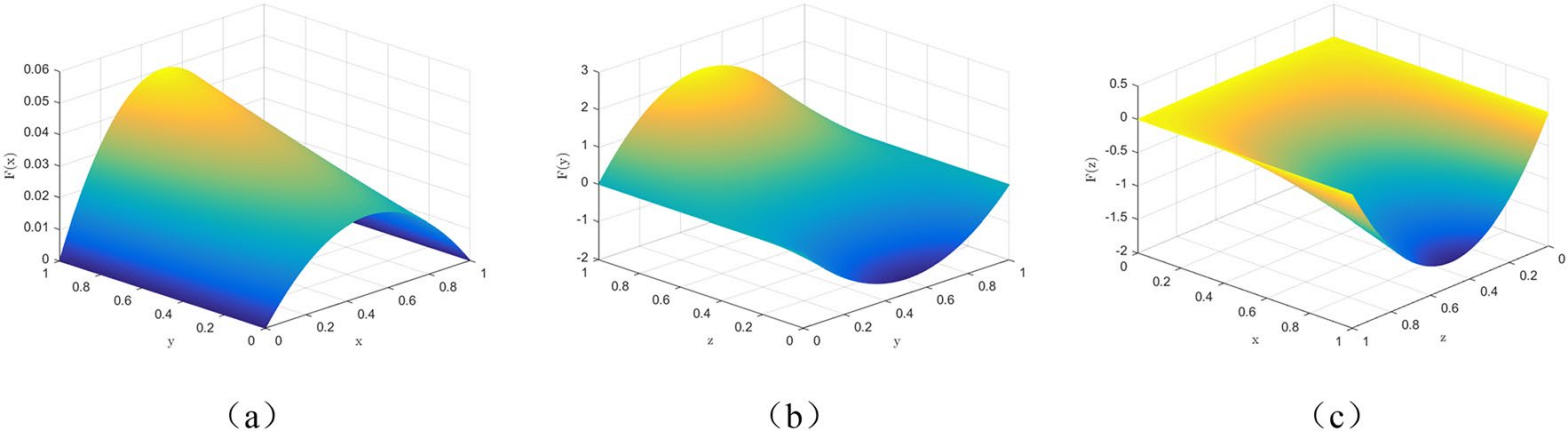
Numerical simulation of single body stability.

Firstly, based on the initial parameters, the replication dynamic equation of the three subjects respectively is used to analyze and assume that the initial value of the third subject is 0.5 when studying the behavior between subjects, and the replication dynamic equation of the social public $F(x)=x(1-x)(0.1y+6.6z-3.2)$, which can be seen from Fig. 5a, regardless of how much $y$ is taken, the image of the function $F(x)$ is a concave function, so ${F}{\prime}\left(1\right)<0$, indicating that $x= 1$ is the stable point, i.e., the social public chooses conscious segregation. The replication dynamic equation of local government is $F(y)=y(1-y)(15z-0.1x-6.3)$, when $z=0.42$, $F(y)=0$, so $z=0.42$ is the critical state. From Fig. 5b, it can be seen that when $z>0.42$, the image of $F(y)$ is a convex function, and ${F}{\prime}\left(1\right)<0$, indicating that $y=1$ is the stable point, i.e., the local government chooses the strict regulation strategy. For $z<0.42$, the image of $F(y)$ is a concave function with ${F}{\prime}\left(0\right)<0$, indicating that $y=0$ is the stable point, i.e., the local government chooses a lax regulatory strategy. Similarly, the replication dynamic equation of the central government sector is $F(z)=z(1-z)(7.6-6.6x-15y)$, when $x=0.015$, $F(z)=0$, so $x=0.015$ is the critical state, as can be seen from Fig. 5c, when $x<0.015$, the image of F(z) is a convex function, ${F}{\prime}\left(1\right)<0$, indicating that $z=1$ is the stable point, i.e., the central government department adopts real-time regulatory strategy; when x > 0.015, the image of $F(z)$ is a concave function and ${F}{\prime}\left(0\right)<0$, indicating that $z=0$ is a stable point, i.e., the central government department adopts a periodic regulatory strategy.

For the public, local government, and central government departments, the stability of individual subjects is consistent with the analysis of Corollary 1, 3, and 5. However, collaborative epidemic prevention and control is a system project. Changes in the behavior of any party will bring about changes in the behavior of other subjects, so the evolutionary path of the tripartite subject system will be simulated numerically below.

### Simulation analysis of the effect of initial willingness analysis on the evolutionary results of the system

**Figure 6:**
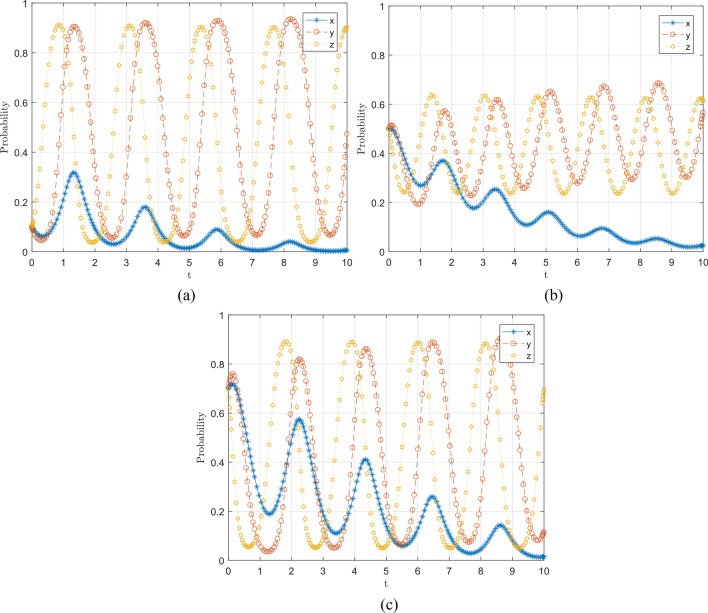
Effect of initial willingness change on evolutionary outcome.

Figure 6 numerical simulation simulates the influence of different initial willingnesss of the social public, local government, and central government departments on the evolutionary results. It can be seen that regardless of the initial willingness of the three subjects of 0.1, 0.5, and 0.7, the social public eventually evolves to 0, and the strategy choices of local government and central government departments are in a fluctuating and unstable state. It means that once the social public chooses to flow freely, it may cause a small outbreak of the epidemic; at this time, the local government and central government departments will strengthen the regulation or punishment, which will produce a more vital constraint on the social public, so the social public will tend to choose conscious isolation when the constraints of the central government departments and local government are more robust because the social public actively cooperates with the government, they cannot obtain the punishment gain, and the government, considering At this time, due to the decline in the level of regulation, the social public will weaken the motivation to take conscious isolation and choose the casual flow strategy considering their interests. It can also be found that the higher the initial willingness of the social public to participate, the slower the convergence rate to the discretionary flow strategy. With the change of initial willingness, the probability of local and central government department strategy choice changes more, but its fluctuation is not controlled.

### Simulation analysis of the impact of changes in key influencing factors on the evolutionary results of the system

**Figure 7:**
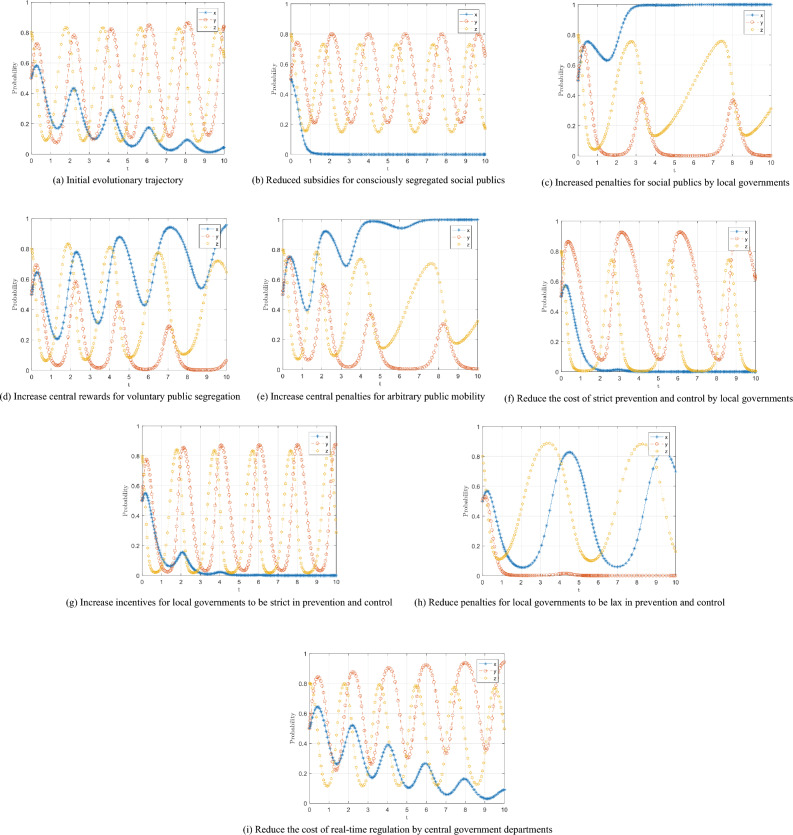
Evolutionary trajectory of the three-party dynamics of the game.

At this stage, the central government department insists on the principle of "whole chain" prevention and control and "dynamic zero," so it is assumed that the central government department is more willing to adopt real-time regulation. In order to avoid the influence of the initial willingness on the evolutionary results, the probability of the initial willingness of the public, local government, and central government is set as (0.5, 0.5, 0.8), and the evolutionary trend of the three parties of the game is shown in Fig. 7a.

It is known by Corollary 2 that the probability of conscious segregation of the social public is positively correlated with the local government's subsidy $Q$, the local government's penalty R for the casual mobility of the social public, the central government department's reward ${M}_{1}$ for the social public, and the central government department's penalty ${N}_{1}$ for the social public, so $Q=1,$ $R=3$, ${M}_{1}=7$, and ${N}_{1}=5$ are adjusted respectively on the basis of the initial parameters to obtain the trend of the three-way dynamic evolution of the game after parameter adjustment, as shown in Figs. 7b–e, respectively.

Comparing Fig. 7a with b, it can be found that although the reduction of subsidies does not change the evolutionary trajectory of the three parties of the game, it can be seen that there is a significant change in the behavior of the social public, and the strategy of the social public changes from fluctuating convergence to a casual mobility strategy to a rapid evolution to a casual mobility strategy within a short period. This indicates that the local government's subsidy to the public is a crucial factor influencing the public's choice of conscious segregation strategy, and the higher the amount of subsidy, the greater the incentive for the public to segregate itself. Comparing Fig. 7(a) with Fig. 7c, we find that increasing the local government's punishment for the social public's casual mobility will tend to choose the conscious segregation strategy. In contrast, the local government's willingness to adopt strict prevention and control decreases because the social public's conscious segregation brings excellent convenience to the local government's prevention and control. The local government cannot gain more punishment benefits from strict prevention and control. Therefore, local governments will gradually choose lenient prevention and control to save on the cost of prevention and control.

Comparing Fig. 7a with d and e, it can be seen that the reward and punishment system of the central government department is an essential means to regulate the social public to adopt the conscious segregation strategy. With the increase of rewards and punishments, the social public finds that the discretionary mobility strategy is no longer the dominant strategy, so they change their strategy to try to choose the conscious segregation strategy, i.e., the higher the amount of rewards and punishments, the higher the expected benefit of the social public to choose the conscious segregation, and the higher the probability of choosing the "conscious segregation" strategy.

It is known by Corollary 4 that the probability of strict prevention and control of local government is negatively correlated with the cost C of strict prevention and control of local government, and positively correlated with the reward ${M}_{2}$ of strict prevention and control of local government by central government department and the penalty ${N}_{2}$ of lenient prevention and control of local government by central government department, so $C=4$, ${M}_{2}=10$ and ${N}_{2}=5$ are adjusted respectively on the basis of the initial parameters to obtain the trend of three-way dynamic evolution of the game after parameter adjustment, as shown in Fig. 7f–h, respectively.

Comparing Fig. 7a and f, we can see that reducing the cost of strict prevention and control of local governments has a more significant impact on the strategy choice of local governments, and the probability of strict prevention and control of local governments increases more obviously and fluctuates more sharply in the game process, therefore, reducing the cost of strict prevention and control of local governments has a direct and noticeable effect on improving strict prevention and control; at the same time, because the probability of strict prevention and control of local governments increases, the probability of central government At the same time, the probability of the central government choosing the "real-time regulation" strategy fluctuates significantly due to the increase in the probability of strict prevention and control by the local government, and there is a significant decrease in the probability of "real-time regulation" compared with Fig. 7a, because the active action of the local government replaces the regulatory behavior of the central government to a certain extent. Similarly, the reward and punishment mechanism of the central government department to the local government can motivate the local government to strictly prevent and control, which is further revealed by the comparison of Fig. 7a and g and h.

**Figure 8:**
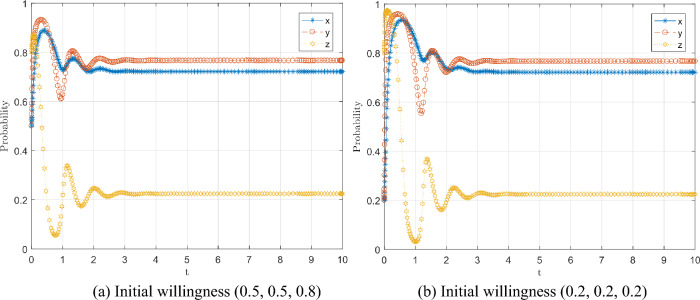
Evolution trend of game agents under dynamic penalty mechanism.

It can be seen through Corollary 6 that the probability of the central government department adopting a real-time regulation strategy is negatively correlated with the cost of real-time regulation ${V}_{1}$, so ${V}_{1}=0.5$ is adjusted on the basis of the initial parameter, and the trend of the three-party dynamic evolution of the game is shown in Fig. 7i. The comparison between Fig. 8a and Fig. 7i reveals that the cost reduction of real-time regulation does not change the fluctuating instability of the central government department and local government. However, the probability of strict prevention and control by local government is increased. The social public's convergence rate to casual flow is similarly slowed down. Reducing real-time regulation costs benefits the central government department for real-time regulation and the compliance of local government and the social public. The probability of both local government and public compliance behavior has increased.

### Stability control strategy

From the above analysis, the strategy choices of the public, local government, and central government departments depend on the game process. Over time, changes in any party's strategy will cause changes in the behavior of other subjects. The instability of such behavior choices will make the behavior of the public and local governments unpredictable, making the real-time supervision by the central government departments challenging to implement effectively, leading to repeated small-scale epidemics or even large-scale outbreaks in the long run. Therefore, controlling the stability of the behavioral choices of the public and local governments is an essential means to ensure that "dynamic zero" is achieved.

The static punishment mechanism, as shown in Figs. 7(e) and 7(h), only changes the size of the fixed value of the parameter. However, it can quickly improve the public and local government's behavior, but as evolution proceeds, static punishment loses its effect. Therefore, the static punishment mechanism is not a stable control strategy of the system because the choice of strategy of the three subjects in the game process is not static but dynamically adjusted; the static punishment mechanism does not make timely adjustments according to the performance behavior of the game subjects, and cannot control the fluctuating instability of the system. This is also why there are often small-scale outbreaks of epidemics under the real-time supervision of central government departments.

Therefore, considering the dynamic punishment mechanism of collaborative prevention and control, the rate of conscious isolation by the public and the rate of strict prevention and control by local governments are associated with the punishment parameters ${N}_{1}, {N}_{2}$, thus introducing the punishment variables ${N}_{1}^{*}, {N}_{2}^{*}$, and the expressions are Eqs. (21) and (22), respectively.

21 $${N}_{1}^{*}=\alpha \left(1-x\right){N}_{1}$$

22 $${N}_{2}^{*}=\beta \left(1-x\right){N}_{2}$$

where α and β are dynamic penalty coefficients, α = 20 and β = 10, respectively, the initial willingness of the game is (0.5, 0.5, 0.8) and (0.2, 0.2, 0.2), and the evolution results are shown in Fig. 8, we can conduct numerical simulation with arbitrary α and β. The simulation results are similar to those shown in Fig. 8. Due to space limitations, we will not repeat them here.

Comparing Fig. 8a with Fig. 7e, h, it can be seen that the dynamic punishment mechanism keeps the volatile instability of the evolving system under control, and the central government department can evolve a higher willingness to isolate consciously and a higher willingness to strictly prevent and control from the public and local government's under a lower willingness to regulate in real-time. Meanwhile, comparing Fig. 8a and b, it can be seen that even though the initial willingness adopted by the three subjects of the game is different, it is still stable in a mixed equilibrium strategy (0.66, 0.73, 0.2), so the dynamic punishment mechanism is the stability control strategy of the system.

## Conclusion and insights

### Research findings

This paper constructs a three-party evolutionary game model among the public, local government, and central government departments based on COVID-19 tripartite subject collaborative prevention and control, solves, derives, and analyzes the influencing factors and evolutionary trajectories of the three actors' collaborative prevention and control, focuses on the influence of essential parameters of collaborative prevention and control and the reward and punishment mechanism on the evolutionary results, and introduces a dynamic punishment mechanism of collaborative prevention and control based on the fact that the static punishment mechanism cannot control the stability of the evolutionary system, and mainly draws the following conclusions:

1. The initial willingness of each subject to participate significantly impacts the promotion of collaborative prevention and control. With the increase of initial willingness to consciously isolate, strictly prevent and control, and real-time supervision strategies, the rate of the social public choosing casual flow strategies slows down, and collaborative prevention and control can be accelerated.
2. The reward and punishment mechanism of the central government department has a positive incentive effect on the local government's strict prevention and control and the social public's conscious isolation. Appropriately increasing rewards, developing reasonable subsidy strategies, and increasing penalties for violations are conducive to the overall optimization of the system, and the punishment mechanism is most sensitive to regulating the discretionary mobility of the public.
3. Government departments' cost of prevention and control can influence their enthusiasm for strict prevention and control and real-time supervision. Reducing the human resources cost, time cost, and financial cost of prevention and control is conducive to government departments performing their duties more responsibly.
4. The static punishment mechanism fails to make timely adjustments according to the strategy choice of each actor. It cannot control the stability of the evolving system. In contrast, the dynamic punishment mechanism considers the punishment parameter to link the casual flow rate with the lenient prevention and control rate, which can effectively control the system's fluctuating instability and is the system's stability control strategy.

### Management insights

The above findings give us insights into the management of public health emergencies in response to:

1. Enhance the protection awareness of the public and local governments in response to public health emergencies, strengthen the determination of the public to isolate consciously, and local governments to prevent and control strictly. In contrast, government departments can appropriately give subsidies to the public who consciously isolate to encourage the public to participate actively in collaborative prevention and control.
2. To improve the government's reward and punishment mechanism, to monitor the behavior of the public and local governments in real-time through the existing technical means, to build a mechanism to push back the responsibility of the "casual flow" type of the public and the "lax prevention and control" type of local governments, to increase the reward for their compliance and the punishment for violations, and to promote the public, local governments and central government departments to respond to public health emergencies in a coordinated manner.
3. Establish and improve the multi-faceted supervision mechanism, enhance the efficiency of government supervision, save the cost of prevention and control, and strengthen the government's enthusiasm in responding to public health emergencies. Give full play to the supervision of the masses, Jitterbug, microblogging, and other new media, and set up a system of rewarding the public for casual mobile reporting to reduce the time and labor costs of government supervision.
4. The implementation of a dynamic punishment mechanism, according to the public health emergencies in response to the main violations of the implementation of dynamic penalties, adhere to the "dynamic zero" principle for the public flow of small-scale epidemic, the local government should pay timely attention to the establishment of the epidemic traceability mechanism, strict prevention, and control, to prevent a large-scale outbreak of the epidemic.

### Research limitations

The main limitation of the governmental reward and punishment mechanism studied in this paper is that the public health emergency prevention and control system is complex. This paper focuses on three subjects, namely, the public, local government, and central government departments, and fails to reflect the situation of other subjects of interest fully; on the other hand, the parameters of the simulation part are set based on real cases, and can only reflect the general situation of the public, local government and central government departments, which has certain limitations (Supplementary Information).

### Supplementary Information

Supplementary Information.

## Data Availability

All data generated or analysed during the course of this study are included in this published article.
